# Sonoporation as an enhancing method for boron neutron capture therapy for squamous cell carcinomas

**DOI:** 10.1186/1748-717X-8-280

**Published:** 2013-12-02

**Authors:** Naofumi Yamatomo, Takaki Iwagami, Itsuro Kato, Shin-Ichiro Masunaga, Yoshinori Sakurai, Soichi Iwai, Mitsuhiro Nakazawa, Koji Ono, Yoshiaki Yura

**Affiliations:** 1 Dental and Oral Surgery, Osaka Saiseikai Senri Hospital, Tsukumodai 1-1, Suita, Osaka, 565-0862, Japan; 2 Department of Oral and Maxillofacial Surgery, Osaka University Graduate School of Dentistry, Yamadaoka 1-8, Suita, Osaka, 565-0871, Japan; 3 Research Reactor Institute, Kyoto University, Kumatori-cho, Sennan-gun, Osaka, 590-0494, Japan

**Keywords:** Ultrasound, Microbubble, Boron neutron capture therapy, Oral squamous cell carcinoma

## Abstract

**Background:**

Boron neutron capture therapy (BNCT) is a selective radiotherapy that is dependent on the accumulation of ^10^B compound in tumors. Low-intensity ultrasound produces a transient pore on cell membranes, sonoporation, which enables extracellular materials to enter cells. The effect of sonoporation on BNCT was examined in oral squamous cell carcinoma (SCC) xenografts in nude mice.

**Materials and methods:**

Tumor-bearing mice were administrated boronophenylalanine (BPA) or boronocaptate sodium (BSH) intraperitoneally. Two hours later, tumors were subjected to sonoporation using microbubbles followed by neutron irradiation.

**Results:**

The ^10^B concentration was higher in tumors treated with sonoporation than in untreated tumors, although the difference was not significant in BPA. When tumors in mice that received BPA intraperitoneally were treated with sonoporation followed by exposure to thermal neutrons, tumor volume was markedly reduced and the survival rate was prolonged. Such enhancements by sonoporation were not observed in mice treated with BSH-mediated BNCT.

**Conclusions:**

These results indicate that sonoporation enhances the efficiency of BPA-mediated BNCT for oral SCC. Sonoporation may modulate the microlocalization of BPA and BSH in tumors and increase their intracellular levels.

## Background

Head and neck cancer, mostly of squamous-cell origin, account for 6.5% of all tumors [1]. The standard treatment for patients with head and neck cancer includes chemotherapeutic drugs, surgery, and radiotherapy [2]. However, the 5-year survival rate of these patients has remained in the range of 50-60% for the last three decades [3]. Radiotherapy plays a key role in the management of locally advanced stages; however, its results remain relatively poor in advanced cases. Several approaches have been developed to improve its efficacy, while maintaining acceptable toxicities.

Boron neutron capture therapy (BNCT) is a binary treatment modality for cancer involving the accumulation of agents containing the isotope ^10^B in cancer cells followed by irradiation with thermal neutrons [4,5]. Capture of a thermal neutron by the ^10^B nucleus initiates a nuclear reaction in which the decay of an excited 10 nucleus produces a high linear energy transfer of alpha-particles and lithium nucleus. These particles have short path lengths of 5-10 μm in water. Side effects typically associated with ionizing radiation can be avoided, if ^10^B agents can be selectively targeted to tumor cells.

Ultrasound-assisted gene delivery has recently been developed as an alternative to a non-viral approach [6,7]. The process referred to as sonoporation is a physical method using an external ultrasound energy source to induce DNA to enter the cell [8,9]. Sonoporation is potentiated by microbubbles, which are stabilized gas microbubbles, originally developed as ultrasound contrast agents for medical imaging [10,11]. Promising results have been reported using sonoporation for gene transfer in various animal tissues and tumor models [10,12-14]. However, the effect of sonoporation on BNCT has not yet been studied. In the present study, we examined whether sonoporation with microbubbles could enhance the antitumor effect of BPA- or BSH-mediated BNCT on human oral squamous cell carcinoma (SCC) xenografts in nude mice.

## Materials and methods

### Cells and tumors

The human oral SCC cell line SAS was obtained from the Japanese Collection of Research Bioresources (Tokyo, Japan). To generate tumors in nude mice, 1 × 10^6^ cells were inoculated subcutaneously into the back of the leg of 5-week-old female Balb/c nude mice (Clea Japan Inc., Japan). Animals were used for BNCT experiments when the tumors reached a suitable size, i.e., approximately 5 mm in diameter.

### Sonoporation

SonoVue (Bracco Diagnostic, Princeton, NJ), a lipid-shelled ultrasound contrast agent filled with perfluorocarbon gas [15,16], is composed of 5 × 10^8^ microbubbles/ml, having an average diameter of 2.5 μm. Microbubbles were used at a dose of 5 × 10^7^ /100 μl. An ultrasound machine, Sonitron 2000 V (NEPAGENE Japan, Chiba, Japan), was used for ultrasound exposure and the head of the transducer, 12 mm in diameter, was placed on the skin through an ultrasound contact gel. The ultrasound frequency was 1 MHz throughout the experiments. Ultrasound was adjusted to supply an intensity of 0.5 W/cm^2^ at a duty cycle of 20% for 10 sec, referring to the results of a previous study [17]. To determine the effect of sonoporation (ultrasound in the presence of microbubbles), tumors in nude mice received microbubbles and then were exposed to ultrasound. Control tumors were left untreated. Tumor length (L) and width (W) were measured every 3-4 days. Tumor volume (V) was determined by the formula: V = LW^2^/2.

To determine the effect of sonoporation with microbubbles on the growth of nude mouse tumor, three tumors in nude mice received microbubbles followed by exposure to ultrasound. They were then followed for 14 days and the tumor size was measured and volume was calculated.

### Boron compounds

^10^B-enriched ( >98%) BPA was obtained from Boron Biologicals, Inc., (Raleigh, NC, USA) and converted to a fructose complex following the method by Coderre et al [18]. The aqueous solution of BPA was prepared at a concentration of 250 mg/ml (21.28 mg ^10^B/ml). BSH (Boron Biologicals) was dissolved in physiological saline. To evaluate their concentrations in tumors, BPA and BSH were injected intarperitoneally at a dose of 250 mg/kg and 75 mg/kg, respectively. For the measurement of ^10^B concentrations, tumors were excised immediately after sonoporation and tumor tissues were dissolved in 0.95 M ammonium sulfate and diluted by 10 volumes of water. The ^10^B concentration in the sample was measured by inductively coupled plasma-mass spectrometry (ICP-MS) Agilent 7700 (Agilent Technologies, Palo Alto, CA, USA). Three to four samples were used to determine the concentrations of ^10^B and the mean values were determined.

### BNCT for oral SCC xenografts in nude mice

Tumor-bearing animals were given an intraperitoneal injection of BPA or BSH. Two hours later, tumors were subjected to sonoporation followed by neutron irradiation. Control tumors were left untreated. Neutron irradiation was delivered via a neutron beam at 1 MW (thermal neutron mode 00-0011) in the Kyoto University Research Reactor (KUR). The mouse was held stationary during the irradiation for 120 min in a custom-designed acrylic resin box from which the tumor-bearing legs were pulled out through a narrow slit and fixed with adhesive. A LiF thermoplastic plate with a hole that defined the irradiation field was placed on the animal to expose the tumor-bearing leg only to the neutron beam. Neutron fluence was measured from the radioactivation of gold foil (3 mm in diameter, 0.05 mm thick) placed at the front and back of the tumors [19]. The average fluence of the thermal neutron was 4.72 × 10^12^ n/cm^2^ at 1 MW. Thermoluminescent dosimeters were used for gamma-ray dosimetry, and the total gamma ray dose was 3.95 Gy after irradiation for 120 min. Thermal neutron fluence was converted to a physical dose using the equation D = Φ{(7.43 × 10^- 14^)B + (6.782 × 10^- 14^)N} + G.

Where D = physical dose, Φ = neutron fluence (n/cm^2^), B = boron concentration (ppm), N = nitrogen concentration (2%), and G = gamma-ray dose (Gy). The ^10^B concentrations were measured twice and the mean values were obtained. The experiments for antitumor effect on BNCT were started at the time of neutron irradiation. Experimental groups included untreated control, neutron only, BPA-mediated BNCT, BPA-mediated BNCT in combination with sonoporation, BSH-mediated BNCT, and BSH-mediated BNCT in combination with sonoporation groups. In the case of BNCT in combination with sonoporation, neutron irradiation was started immediately after sonoporation. Four tumors in each group were used in an experiment. Repeated data were combined to evaluate tumor volume and survival rate. Experiments using nude mice were performed with the approval of the Institute of Laboratory Animals, Osaka University Graduate School of Dentistry.

### Histological examination

Tumors were treated by BNCT in combination with sonoporation and subjected to histological examination 24 h later. Tumor tissues fixed in 10% formalin were embedded in paraffin. Sections were deparaffinized, rehydrated, and stained with hematoxylin and eosin (H-E).

### Statistical analysis

All values were expressed as the mean ± SD. A repeated measure ANOVA test was used to determine the significance of differences in tumor volume. After confirming significant differences, the mean tumor volume in each group was analyzed with the unpaired Student’s *t* test. The survival time of animals was converted to Kaplan-Meier plots, and the significance of the difference in survival was calculated using the generalized Log-rank test. These statistical analyses were performed using the software Statcel2, version 2 (OMS, Tokyo, Japan). A value of P < 0.05 was considered to be significant.

## Results

### Incorporation of ^10^B into oral SCC xenografts in nude mice

In previous studies, the ^10^B concentration of tumors reached a maximal level 2 h following the intraperitoneal injection of BPA [20]. Tumor-bearing mice were also intraperitoneally administered BPA or BSH and tumors were removed 2 h later to measure concentrations of ^10^B. In the sonoporation groups, tumors received microbubbles, and were then exposed to ultrasound prior to sampling for the measurement of intratumoral ^10^B concentrations. When tumors that received intraperitoneal injection of BPA were subjected to sonoporation, the ^10^B concentration was increased from 16.82 ± 6.23 to 22.72 ± 7.71 ppm. In BSH-injected animals, the ^10^B concentration in tumors was increased from 3.18 ± 1.09 to 9.84 ± 7.53 ppm by sonoporation (Table 1). Using ^10^B concentrations in the tumors, the physical doses in the BPA-mediated BNCT and BPA-mediated BNCT in combination with sonoporation groups were calculated as 10.49 ± 2.18 and 12.55 ± 2.70 Gy, respectively. Physical doses in the BSH-mediated BNCT and BSH-mediated BNCT in combination with sonoporation groups were 5.70 ± 0.38 and 8.04 ± 2.64 Gy, respectively (Table 1). In both boron concentration and physical doses, there was a significant difference (P < 0.05) between BSH groups with or without sonoporation, but such difference was not observed in BPA.

**Table 1:** Physical doses for nude mouse tumors

**Group**	^**10**^**B concentration (ppm)**	**Physical dose (Gy)**
N	0	4.59
BPA + N	16.82 ± 6.23	10.49 ± 2.18
BPA + S + N	22.72 ± 7.71	12.55 ± 2.70
BSH + N	3.18 ± 1.09	5.70 ± 0.38
BSH + S + N	9.84 ± 7.53*	8.04 ± 2.64*

*N*, neutron irradiation; *BPA*, Intraperitoneal injection of BPA; *S*, Sonoporation (ultrasound irradiation in the presence of microbubbles); *BSH*, Intraperitoneal injection of BSH. Values except for N were means ± SD of three to four determinations. *P < 0.05 vs. BSH + N.

### Effect of sonoporation on tumor volume

Whether sonoporation with microbubbles could affect the growth of nude mouse tumor was examined. Tumors received microbubbles followed by exposure to ultrasound. Fourteen days later, the tumor volume was increased from 242 ± 96 mm^3^ to 1286 ± 557 mm^3^ and was 5.3-fold higher than that of the initial volume, while tumor volume in the control was 252 ± 87 mm^3^ to 1359 ± 380 mm^3^ and the increase was 5.4-fold. No significant difference was observed between the groups treated with sonoporation or controls, which indicated that ultrasound with microbubbles did not inhibit the growth of tumors.

### Effect of BNCT in combination with sonoporation on tumor volume

**Figure 1:**
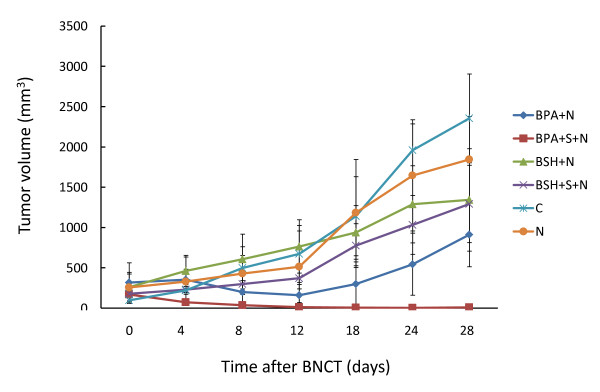
**Effect of BNCT in combination with sonoporation on tumor volume.** Nude mice carrying SAS tumors received BPA or BSH intraperitoneally. Two hours later, tumors were treated with sonoporation followed by neutron exposure. The experimental groups were as follows: untreated control (C), neutron only (N), BPA-mediated BNCT (BPA + N), BPA-mediated BNCT in combination with sonoporation (BPA + S + N), BSH-mediated BNCT (BSH + N), and BSH-mediated BNCT in combination with sonoporation (BSH + S + N). After BNCT, tumor size was measured during the experiment and tumor volume was determined. Four tumors in each group were used in an experiment. Repeated data were combined to evaluate tumor volume and survival rate. Data were means ± SD.

Tumor-bearing animals were administered BPA or BSH intraperitoneally. Two hours later, tumors were treated with BNCT in combination with sonoporation or without. The experimental groups were as follows: untreated control, neutron only, BPA-mediated BNCT, BPA-mediated BNCT in combination with sonoporation, BSH-mediated BNCT, and BSH-mediated BNCT in combination with sonoporation. In control animals, tumors continued to grow and were 2354 ± 551 mm^3^ 28 days after the start of the experiment (Figure 1). A significant difference was observed between the BPA-mediated BNCT and neutron only groups 28 days after BNCT (P < 0.05). When tumors were subjected to BPA-mediated BNCT in combination with sonoporation, tumor volume was markedly decreased from 4 days after neutron irradiation and most tumors became undetectable at 8 days. A significant decrease was observed between the BPA-mediated BNCT and BPA-mediated BNCT in combination with sonoporation groups 28 days after BNCT (p < 0.01). In the BSH-mediated BNCT group, sonoporation did not alter the effect of BNCT on tumor volume (Figure 1).

**Figure 2:**
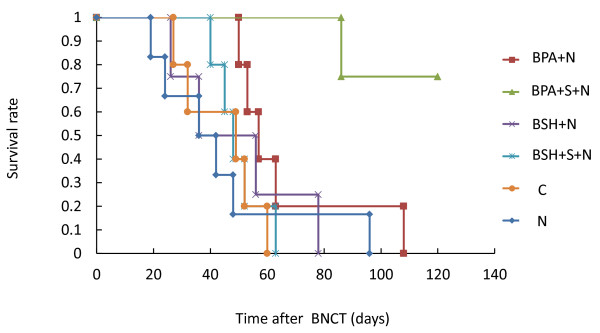
**Effect of BNCT in combination with sonoporation on the survival rates of animals.** Nude mice carrying SAS tumors were treated as described in Figure 1. After the treatment, animals were observed and their survival rates were measured.

All untreated control animals died within 60 days of the start of the experiment. Animals exposed to neutron only, BSH-mediated BNCT, BSH-mediated BNCT in combination with sonoporation, and BPA-mediated BNCT died by day 96, 79, 62, and 109, respectively. However, animals whose tumors were treated with BPA-mediated BNCT in combination with sonoporation survived longer than those in other groups (Figure 2). The survival rate of mice treated with BPA-mediated BNCT was significantly different from that of mice treated with BPA-mediated BNCT in combination with sonoporation (P < 0.05).

**Figure 3:**
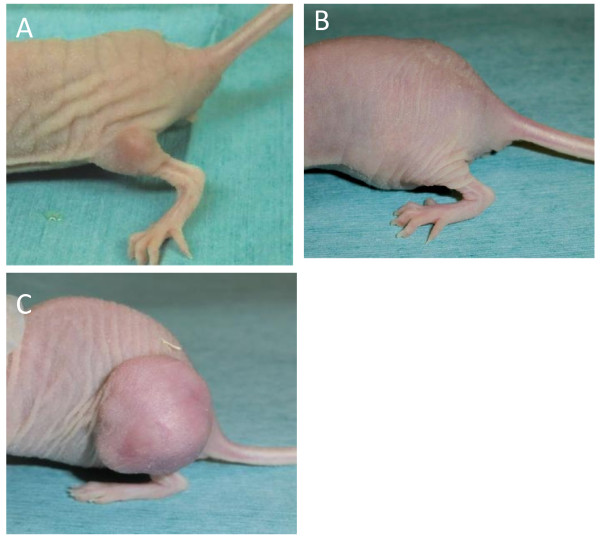
**Macroscopic observation of tumors treated with BNCT. (A)** SAS cells were inoculated into the leg of nude mouse subcutaneously and tumors were produced in 7 days. **(B)** When treated with BPA-mediated BNCT in combination with sonoporation after the intraperitoneal injection of BPA, tumors became undetectable 18 days later. The experiments for antitumor effect on BNCT were started at the time of neutron irradiation. **(C)** Untreated tumors 18 days from the start of the experiment showed extensive growth.

The skin on tumors had a normal appearance during the experiment following BPA-mediated BNCT in combination with sonoporation (Figure 3). No ulceration was observed in any experimental animal, until the extensive growth of tumors and necrotic changes occurred.

### Histological examination of BNCT-treated tumors

**Figure 4:**
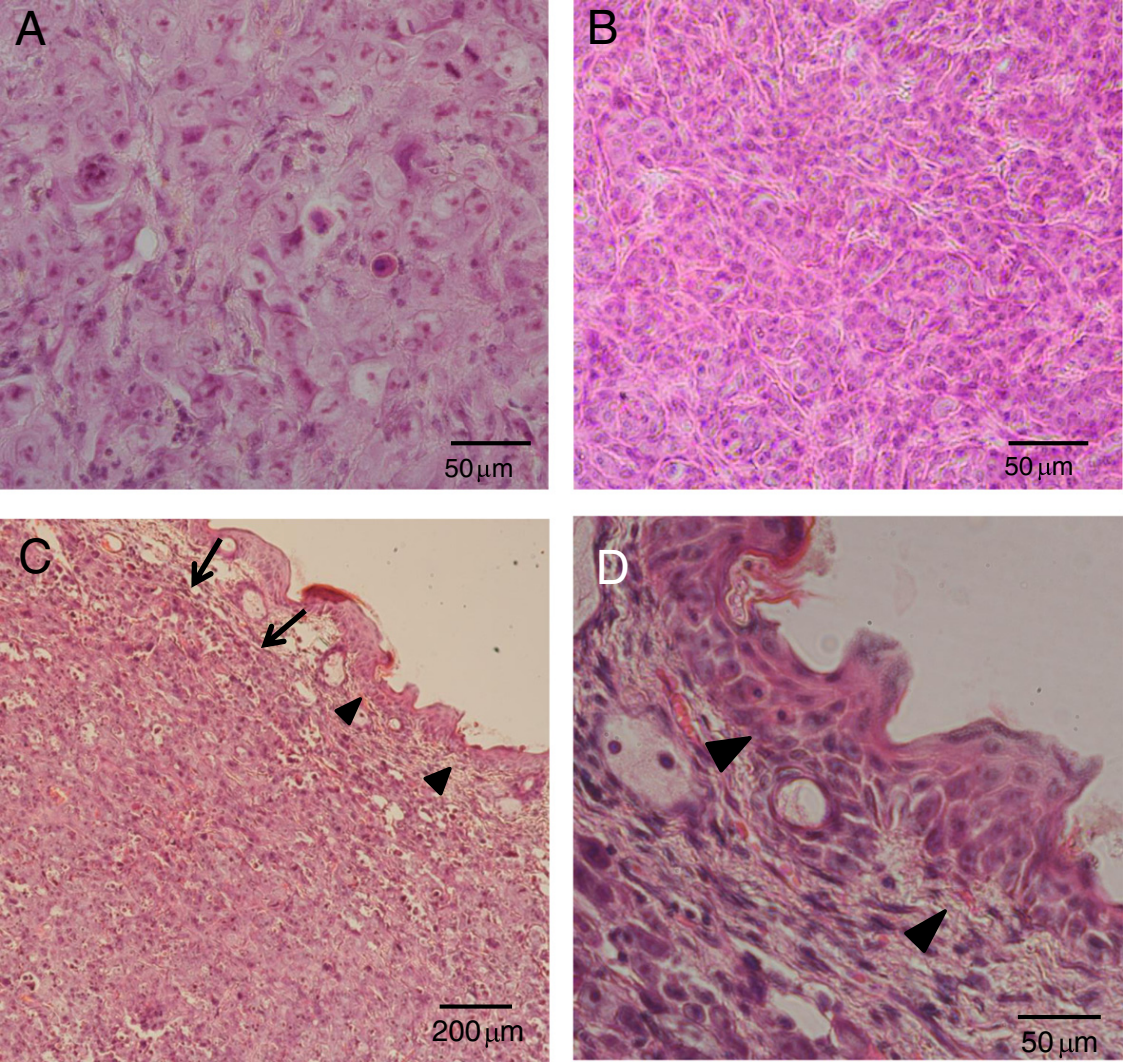
**Histological examination of tumors.** Nude mouse tumors in the untreated control group **(A)** and BPA-mediated BNCT in combination with sonoporation group **(B, C and D)** were subjected to histological examinations. Twenty four hours after treatment, the vacuolation of tumor cells, fragmentation of nuclei, enlarged and multinucleated cells were observed **(B)**. Although vacuolation appeared in the dermis, cell layers in the epidermis (arrow head) were preserved without degradation **(C and D)**. Arrows showed the margin of the tumors produced by subcutaneous inoculation of SAS cells **(C)**.

BNCT-treated and untreated tumors were subjected to histological examination. In control tumors, polyhedral cells had intimate interactions each other, forming a solid mass (Figure 4A), and were separated from the covering skin epidermis by connective tissue. When tumors were treated with BPA-mediated BNCT in combination with sonoporation and were observed 24 h later, tumor nests became unclear. Vacuolation of the cytoplasm, fragmentation of nuclei and multinuclear cells were observed (Figure 4B). The structure of the covering skin was preserved and the degradation of basal cells was not observed (Figure 4C, D).

## Discussion

Since BPA is an analog of phenylalanine, its uptake has been associated with amino acid synthesis in cells and the cell cycle [21-23]. The uptake of BPA has also been shown to be dependent on amino acid transporters such as L-type amino acid transporter 1 (LAT-1) and LAT-4 [24,25]. LAT-negative cells may survive BNCT and cause recurrence. The active transport of BSH has been shown to not occur [20]. Thus, techniques to improve intracellular ^10^B concentrations are required.

Electroporation is one of the methods used to increase the delivery of exogenous molecules into cells and has been shown to be effective in the introduction of drugs, DNA, proteins, dyes, and other molecules into cells both *in vitro* and *in vivo*[26]. The application of electric pulses increased and prolonged the entrapment of BSH in B16F1 melanoma tumors [27]. However, it requires invasive needle electrode placement into the target tissue to deliver electric pulses. Skin abrasion may be a complication of this procedure. Sonoporation with low intensity ultrasound is another technique used to stimulate cell membrane permeabilization and allow the incorporation of extracellular materials. It offers advantages over electroporation, primarily as a result of its relatively non-invasive nature [14]. The intensity of ultrasound is within the range of 0.5 to 3 W/cm^2^ and is appropriate for medical applications. In the presence of microbubbles, its efficiency on permeability of the cell membrane was shown to markedly increase [11]. Accordingly, an enhancement in the antitumor effect of chemotherapeutic agents and the delivery of plasmid DNA *in vitro* and *in vivo* can be achieved [12-14]. Li et al. [13] infected human retinal pigmented epithelial cells with adeno-associated virus under the following ultrasound conditions: 1.0 W/cm^2^, a duty cycle of 20%, and an exposure period of 20 sec, and found an increase in the green fluorescent protein-positive rate from 17 to 32%. No damage to cultured cells was observed under our ultrasound conditions of an intensity of 1 W/cm^2^ and 20% duty cycle for 10 sec [17]. The combination of ultrasound with microbubbles did not inhibit the growth pattern of oral SCC xenografts. Thus, it is unlikely that sonoporation affects the growth of tumors.

We previously reported that the ^10^B concentration in tumors peaked at approximately 2 h after an intraperitoneal injection of BPA, while BSH did not show a time-dependent increase in ^10^B concentrations in tumors [20]. In the present study, we performed sonoporation on tumors 2 h after the intraperitoneal administration of BPA, and found that the ^10^B concentration in tumors was slightly increased. The highest concentration was obtained in the BPA-mediated BNCT group. The effect of sonoporation on ^10^B concentration was more prominent in BSH-administered animals than in BPA-administered animals, although the basal level of BSH was low. It is likely that sonoporation can enhance the concentrations of BPA and BSH in nude mouse tumors and also the physical doses by neutron irradiation, although the effect on BPA is not remarkable.

Consistent with the results of ^10^B concentrations achieved in tumors, tumor growth was suppressed more by BPA-mediated BNCT than neutrons only, however, complete remission was not achieved at this physical dose. We found that the increase in intratumoral ^10^B concentrations by sonoporation was 1.4-fold. Nevertheless, the growth of tumors was markedly suppressed, and most tumors became undetectable 12 days after neutron exposure. Although the amounts of BPA in the tumors did not increase significantly, sonoporation may affect the microlocalization of BPA in tumors and promote the incorporation of BPA into each tumor cell. This contributed to the enhancement in antitumor ability of BPA-mediated BNCT. The BPA levels in quiescent tumor cells and low-LAT-expressing tumor cells may be also improved by sonoporation. Regarding to BSH, sonoporation did not enhance the antitumoral effect of BNCT, probably, which may have been because the concentration of ^10^B attained in tumors was very low. Recent studies indicated that BSH could be a source for novel forms of ^10^B compounds such as BSH-encapsulating liposomes, and transferrin-conjugated polyethylenglycol liposomes encapsulating BSH [28,29]. The effect of sonoporation should be investigated using novel BSH derivatives.

The area exposed to ultrasound was defined by the diameter of the ultrasound transducer. We used a transducer 12 mm in diameter, which completely covered tumor surface, and found a significant effect of BPA-mediated BNCT. An appropriate transducer should be used to enhance the effect of BNCT and reduce damage to the surrounding tissues. If sonoporation increased the incorporation of BPA into the skin, BNCT may affect its viability. However, no apparent skin damage occurred at the site of sonoporation and cell layers of the epidermis did not degrade histologically (Figures 3 and 4). The walls of tumor blood vessels were previously shown to have widened interendothelial junctions and a discontinuous or absent basement membrane, which made tumor vessels leaky [30]. Although aberrant tumor blood vessels allow BPA to reach each tumor cells, this distribution of BPA does not occur in the normal epidermis. More importantly, the expression of LAT is increased in many types of tumor cells [26]. In this situation, the skin may avoid damage by BNCT, even when sonoporation is applied.

## Conclusions

We demonstrated that sonoporation increased the incorporation of BPA and BSH into oral SCC xenografts in nude mice. The growth of tumors was more suppressed by BPA-mediated BNCT in combination with sonoporation than by BPA-mediated BNCT and the survival rate was also prolonged. This method may be useful for enhancing the antitumor effect of BPA-mediated BNCT.

## Competing interests

The authors declare that they have no competing interests.

## Authors’ contributions

NY and TI carried out the experiments. IK provided the compounds and carried out the experiments. SM, YS, and KO participated in designing the reactor. SI and MN participated in sonoporation and the histological study. YY conceived the study and anticipated its design and coordination. All authors read and approved the final manuscript.
